# The effect of peer mentoring program on clinical academic progress and psychological characteristics of operating room students: a parallel randomized controlled trial

**DOI:** 10.1186/s12909-024-05424-z

**Published:** 2024-04-22

**Authors:** Amin Sedigh, Sara Bagheri, Pariya Naeimi, Vahid Rahmanian, Nader Sharifi

**Affiliations:** 1 Molecular and Medicine Research Center, Khomein University of Medical Sciences, Khomein, Iran; 2 Department of Medical Education, School of Medical Education and Learning Technologies, Shahid Beheshti University of Medical Sciences, Tehran, Iran; 3 Student Research Committee, Khomein University of Medical Sciences, Khomein, Iran; 4 Department of Public Health, Torbat Jam Faculty of Medical Sciences, Torbat Jam, Iran; 5 Department of Public Health, Khomein University of Medical Sciences, Khomein, Iran

**Keywords:** Education, Mentors, Operating Room, Stress, Students

## Abstract

**Background:**

One of the new educational systems is the mentorship method. This study aimed to investigate the effect of peer mentoring program on clinical academic progress and psychological characteristics of operating room students.

**Methods:**

This research was a randomized controlled trial that was conducted on undergraduate students in the operating room department of Khomein Faculty of Medical Sciences, Markazi Province in Iran. The number of operating room students were 70 that were divided into intervention and control groups by random allocation using Permuted Block Randomization. Inclusion criteria included all operating room students who were in internship, and exclusion criteria included failure to complete the questionnaires. The data collection tools were the demographic questionnaire, Depression Anxiety Stress Scale, Rosenberg Self-Esteem Scale and Situational Motivational Scale. In the control group, clinical training was done in the traditional way. In the intervention group, training was done by peer mentoring method. The obtained data were analyzed using descriptive statistics, independent t-test, paired t-test, chi-square test, ANCOVA, univariable and multivariable linear regression.

**Results:**

The study revealed significant differences between the intervention and control groups. Post-intervention, the intervention group demonstrated substantial increases in self-confidence (mean difference = 5.97, *p* < 0.001) and significant reductions in stress levels (mean difference = -3.22, *p* < 0.001). Conversely, minimal changes were noted in the control group for both self-confidence (mean difference = 0.057, *p* = 0.934) and stress levels (mean difference = 0.142, *p* = 0.656). Although both groups experienced decreases in anxiety and depression levels, these changes were not statistically significant (*p* > 0.05). Furthermore, the intervention significantly enhanced academic progress in the intervention group compared to the control group (mean difference = 20.31, *p* < 0.001).

**Conclusion:**

The results showed that the implementation of the peer mentoring program was effective in improving academic progress, self-confidence, and reducing the stress of operating room students. Therefore, this educational method can be used in addition to the usual methods to improve the education of operating room students.

## Introduction

Using effective training methods can increase people's motivation and commitment, increase productivity and reduce mistakes [1]. Clinical training is an important part of training in medical sciences, which plays an essential role in shaping the basic skills and professional abilities of students, including students of the operating room [2, 3]. Learning and mastering work roles and tasks in the operating room environment is challenging; In addition, operating room students should be trained in many interventions in the surgical process before, during and after surgery [4].

Operating room students are affected by various stresses during the course of clinical training, and various contextual and environmental factors play a role in creating this stress [5]. The results of a study among nursing students showed the prevalence of depression, anxiety and stress symptoms to be 28.7%, 41.7% and 20.2%, respectively [6]. Also, studies have shown students' self-efficacy at an average level [7]. The experience of stress in the clinical environment can affect students' learning and acquisition of clinical skills and lead to a drop in their academic performance [8, 9]. Considering the high level of stress and the fact that mistakes have no place in the operating room, it is important to pay attention to the quality of training of operating room students and to strengthen the knowledge and skills of future operating room personnel [10].

Learners and students prefer new educational methods to traditional and passive methods. Active approach is a form of teacher-learner interaction in which learners are no longer passive listeners, but active participants in the learning process [11, 12]. The basis of active and comprehensive learning methods is that learning is based on experience and learners actively create knowledge based on their personal experience [13–15]. The importance of active learning has led professional associations and accreditation organizations, as well as organizations such as UNESCO, to recommend active learning methods in education [16].

One of the new educational systems is the mentorship method. In this educational method, the mentor and mentee establish a long-term relationship based on friendship with each other. Positive attitude, experience and volunteering are characteristics of mentorship [17, 18]. For the first time, Whitman and Fife examined the peer teaching strategy in university education. In this method, higher year students teach practical and theoretical lessons to lower year students [19, 20]. The implementation of the mentorship program increases self-confidence, emotional support, and increases students' interactions [21, 22]. When students, despite having knowledge and ability in clinical practice, lack sufficient competence, the reason may be a lack of self-confidence, confidence in their own ability, or understanding of the necessary self-efficacy [23, 24]. This study was conducted with the aim of investigating the effect of peer mentoring program on clinical academic progress and psychological characteristics of operating room students.

## Method

### Study design

This research was a parallel randomized controlled trial that was conducted on undergraduate students in the operating room department of Khomein Faculty of Medical Sciences, Markazi Province in Iran from September 2022 to April 2023.

### Participants

The number of operating room students were 70, who were included in the study by census method. Inclusion criteria included all operating room students who were in internship, and exclusion criteria included failure to complete the questionnaires.

### Randomization and blindness

**Fig. 1:**
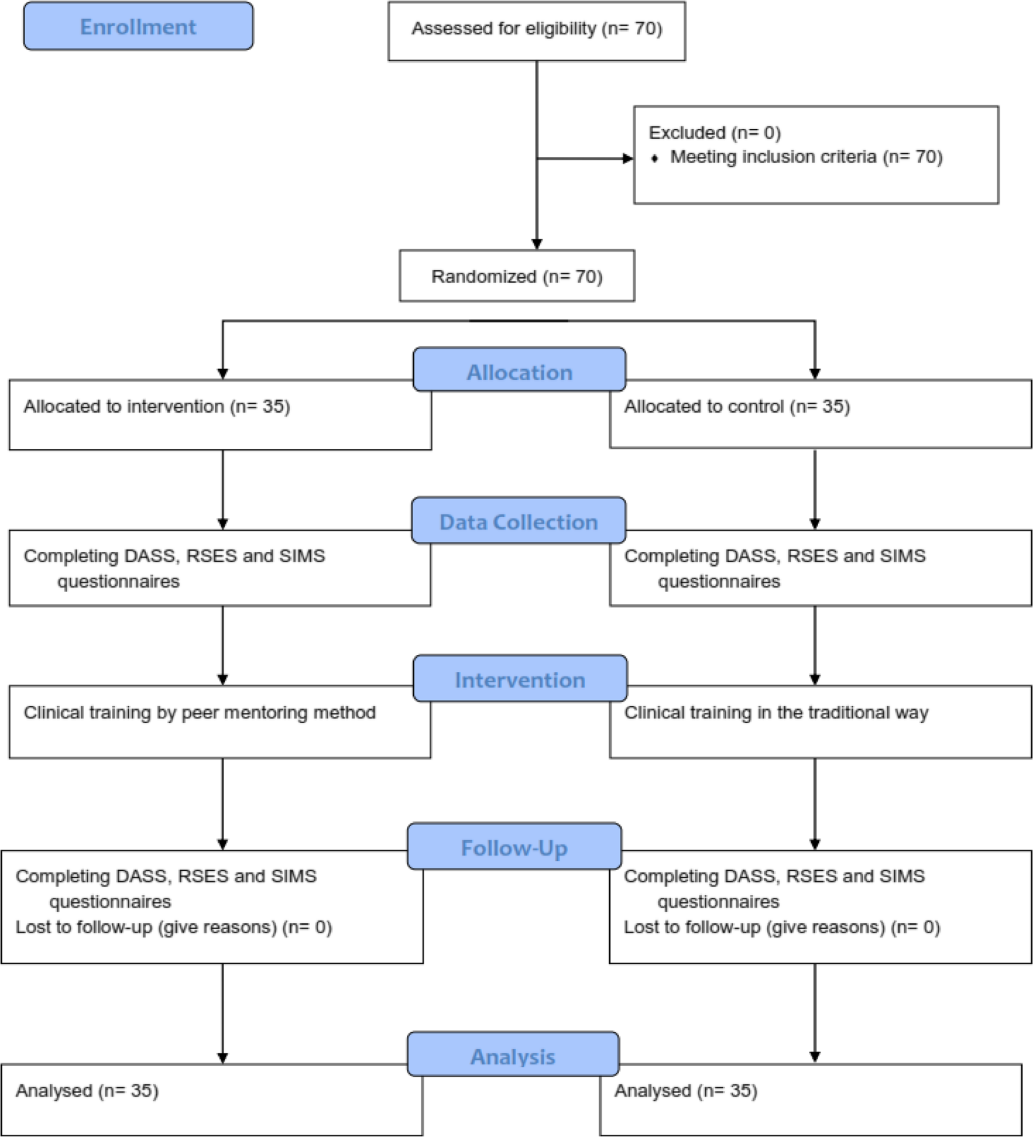
Consort -flow- diagram

First, the students completed the written consent to participate in the study, and then they were divided into intervention and control groups by random allocation using Permuted Block Randomization [25]. Therefore, 35 participants were placed in each group. Then the participants of the intervention and control groups completed the questionnaires before the beginning of the internship. Due to the nature of the intervention in the present study, it was not possible to blind the subjects under the study. Therefore, blinding was performed on those who collected and recorded the data and those who performed the analysis. This research was designed and implemented according to the CONSORT guidelines (Fig. 1).

### Instrument and data collection

The demographic questionnaire included gender, age, marital status, economic status of the family, education level of parents and occupation of parents.

Depression Anxiety Stress Scale (DASS) consists of three subscales including 7 questions for each. Each question is scored from 0 (does not apply to me at all) to 3 (completely applies to me). Each of the areas of stress, anxiety and depression has 7 questions and the minimum score for each area is 0 and the maximum score is 21. The score of each area is obtained from the sum of the scores of the answers given to the questions of that area. Antony et al. analyzed the mentioned scale; The results of the correlation calculation indicated a correlation coefficient of 0.48 between the two factors of depression and stress, a correlation coefficient of 0.53 between anxiety and stress, and a correlation coefficient of 0.28 between anxiety and depression [26]. The reliability of this scale in Iran in a sample of 400 participants was reported as 0.7 for depression, 0.66 for anxiety and 0.76 for stress [27]. Also, in the validation study of this questionnaire in Iran by Sahebi et al. the reliability of this scale was investigated through internal consistency and its validity using factor analysis and criterion validity with the simultaneous implementation of Beck depression, Zang anxiety and perceived stress tests. In general, the obtained reliability and validity coefficients were very satisfactory and significant at the *p* < 0.001 level. The correlations between DASS depression subscale with Beck depression test were 0.70, DASS anxiety subscale with Zang anxiety test was 0.67, and DASS stress subscale with perceived stress test was 0.49. The internal consistency of DASS scales was also calculated using Cronbach's alpha and these results were obtained: depression 0.77, anxiety 0.79 and stress 0.78 [28].

Rosenberg Self-Esteem Scale (RSES) consists of 10 two-choice questions. Every statement that applies to the person receives the answer "I agree" and every statement that does not apply to the person receives the answer "I disagree". A positive answer to each of statements 1 to 5 will receive a positive score of one, a negative response to statements 1 to 5 will receive a negative score of one, a positive response to statements 6 to 10 will receive a negative score of one, and a negative response to statements 6 to 10 will receive a positive score of one. Then the total score is calculated. A positive score of 10 indicates the highest level of self-esteem, and a negative score of 10 indicates very low self-esteem. The retest correlation is in the range of 0.82–0.88 and the internal consistency coefficient or Cronbach's alpha is in the range of 0.77–0.88, this scale has satisfactory validity (0.77). It also has a high correlation with the New York and Guttman National Questionnaire in measuring self-esteem, so its content validity is also confirmed [29]. In Iran, Cronbach's alpha coefficients of 0.84 to 0.92 have been reported for this scale. Also, the reliability and validity of this tool has been checked by factor analysis, dichotomization and re-sampling methods, and the results show that this scale can be used in Iran as well [30].

The Situational Motivational Scale (SIMS): After confirming the content validity of the tool in Iran, its reliability has been confirmed by retest method (73.76) and Cronbach's alpha has been reported as 74–88%. The short form of this questionnaire was made by Bahrani in Shiraz. This questionnaire has 49 statements that are arranged on a Likert scale from completely disagree [1] to completely agree [5]. Reliability of the 49-question questionnaire used in this research was measured by Bahrani by retesting and calculating Cronbach's alpha. In the retest method, the reliability coefficient of the whole test was 0.95. Also, the internal consistency of the questionnaire was calculated as 0.77 [31, 32].

### Intervention program

In the control group, clinical training was done in the traditional way with the help of a trainer. In the intervention group, training was done by peer mentoring method with the help of fourth year operating room students and under the supervision of the instructor. Based on the overall GPA criteria, the first to sixth ranked students were selected as mentor students. Before using the students as mentors in the internship, 3 training sessions were held for them by the professors of the operating room.

In these meetings, the lesson plan of the internship course was fully explained based on the last chapter of the operating room field, and the necessary points regarding training and how to deal with students were explained.

Then, these students participated in three tests and the first to third students of each test were selected as mentors. Therefore, a total of nine students were selected as mentors. In the intervention group, internship training was carried out with the implementation of peer mentoring program during one academic semester. Students of the intervention group (35 participants) were placed in five groups of seven according to the internship program. The total training sessions of each group were 18 sessions, nine of which were conducted by the method of peer mentoring program. A total of 45 peer mentoring sessions were conducted for all groups. Each of the mentors mentored a seven-person group of mentees during nine sessions. At the beginning of each session, the mentor briefly explained the topics to the mentees according to the educational topics and guided them practically during the session. It should be noted that all the meetings were held under the supervision of the main teacher of the course and if necessary, this person provided the necessary guidance.

At the end of the academic semester, the Depression Anxiety Stress Scale, Rosenberg Self-Esteem Scale (RSES) and Situational Motivational Scale (SIMS) were completed again by the students of the intervention and control groups.

### Statistical analysis

Stata software version 14 was used for the data analysis process. Initially, the data's normality was verified using the Kolmogorov–Smirnov test. The results were presented as mean, standard deviation, frequency, and percentage in the section on descriptive statistics.

The means of the study variable between the intervention and control groups were compared using an independent t-test, and the means before and after the intervention were compared using a paired t-test in the analytical statistics section. The Chi-square test was used to compare the associations between qualitative variables in the various groups.

The ANCOVA test was conducted after the intervention to control for any baseline differences in scores of self-confidence, stress, perceived anxiety, depression and academic progress between the two groups before the intervention (pre-test). This adjustment was made to account for any potential confounding factors that may have influenced the outcomes.

Univariable and multivariable linear regression by the backward method was applied to examine the association between self-confidence, stress, perceived anxiety, depression, gender, mother's education, father's education, family economic, and academic progress. A significance threshold of less than 0.05 was used.

## Results

The mean age of participants was 22.31 ± 2.59. Thirty-six individuals (51.4%) were female, and 50 individuals (71.4%) were single. Regarding education, 22 participants (31.4%) held diplomas from their fathers, and 21 participants (30%) held diplomas from their mothers. In terms of mothers' occupations, 35 individuals (52.9%) were housewives, and 31 individuals (44.3%) reported their family's economic status as medium (Table 1). On the other hand, there were no significant differences in age, gender, marital status, mothers' education, fathers' education, fathers' occupation, mothers' occupation, and family economic status between the intervention and control groups(*p* > 0.05) (Table 1). Also, in terms of variables of self-confidence, stress, anxiety, depression and academic progress between the intervention and control groups, no significant difference was observed before the intervention (*p* > 0.05) (Table 2).

**Table 1:** Comparison of demographic variables in intervention and control groups before educational intervention

Variable	Category	Total (*n* = 70)	Intervention group (*n* = 35)	Control group (*n* = 35)	*P*-value
Age(yr), mean (SD)	NA	22.31(2.59)	22.54(2.98)	22.08(2.16)	0.783^a^
Gender, n (%)	Male	34(48.6)	17(48.6)	17(48.5)	0.594^b^
	Female	36(51.4)	18(51.4)	18(51.4)	
Marital status, n (%)	Single	50(71.4)	25(71.4)	25(71.4)	0.604^b^
	Married	20(28.6)	10(28.6)	10(28.6)	
Mother's education, n (%)	Illiterate	15(21.4)	9(25.7)	6(17.1)	0.255^b^
	Elementary	15(21.4)	9(25.7)	6(17.1)	
	Secondary	10(14.3)	3(8.6)	7(20)	
	Diploma	22(31.4)	11(31.4)	11(31.4)	
	University	8(11.4)	3(8.6)	5(14.3)	
Father's education, n (%)	Illiterate	15(21.4)	9(25.7)	6(17.1)	0.911^b^
	Elementary	14(20)	5(14.3)	9(25.7)	
	Secondary	7(10)	4(11.4)	3(8.6)	
	Diploma	21(30)	11(31.4)	10(28.6)	
	University	13(18.6)	6(17.1)	7(20)	
Mother's occupation, n (%)	Housewife	37(52.9)	17(48.6)	20(57.1)	0.419^b^
	Azad	6(8.6)	3(8.6)	3(8.6)	
	worker	6(8.6)	3(8.6)	3(8.6)	
	Employee	19(27.1)	11(31.4)	8(22.9)	
	Retired	2(2.9)	1(2.9)	1(2.9)	
Father's occupation, n (%)	Unemployed	4(5.7)	3(8.6)	1(2.9)	0.734^b^
	Azad	30(42.9)	14(40)	16(45.7)	
	worker	17(24.3)	7(20)	10(28.6)	
	Employee	17(24.3)	10(28.6)	7(20)	
	Retired	2(2.9)	1(2.9)	1(2.9)	
Family economic, n (%)	Weak	18(25.7)	8(22.9)	10(28.6)	0.633^b^
	Medium	31(44.3)	16(45.7)	15(42.9)	
	Good	21(30)	11(31.4)	10(28.6)	

*SD* Standard deviation, *NA* Not applicable

^a^Independent t test

^b^Chi-square test, Significance level < 0.05

**Table 2:** Comparison of self-confidence, stress, anxiety, depression and academic progress in two groups of intervention and control group

Variable		Before Intervention Mean ± SD	After Intervention Mean ± SD	Mean difference	*p*-value*
Self-confidence	Intervention group	1.31 ± 2.66	7.28 ± 2.16	5.97 ± 3.03	** < 0.001**
	Control group	1.22 ± 2.67	1.28 ± 2.67	0.057 ± 4.02	0.934
	*p*-value**	0.894	** < 0.001**	** < 0.001**	
DASS Stress	Intervention group	12.65 ± 1.67	9.24 ± 1.75	-3.22 ± 2.19	** < 0.001**
	Control group	12.25 ± 1.25	12.40 ± 1.24	0.142 ± 1.88	0.656
	*p*-value**	0.316	** < 0.001**	** < 0.001**	
DASS Anxiety	Intervention group	11.34 ± 1.90	10.51 ± 2.03	-0.80 ± 2.68	0.087
	Control group	11.02 ± 2.28	10.71 ± 2.09	-0.31 ± 2.76	0.506
	*p*-value**	0.572	0.687	0.459	
DASS Depression	Intervention group	10.08 ± 1.66	9.37 ± 1.98	-0.71 ± 2.49	0.099
	Control group	10.42 ± 1.09	10.05 ± 1.90	-0.37 ± 2.11	0.306
	*p*-value**	0.313	0.146	0.537	
Academic progress	Intervention group	83.40 ± 18.35	103.71 ± 15.92	20.31 ± 22.61	** < 0.001**
	Control group	80.42 ± 22.60	81.65 ± 22.66	1.22 ± 32.62	0.825
	*p*-value**	0.548	** < 0.001**	**0.006**	

*SD* Standard deviation

**Independent t test, *Paired t-test, “- “Not applicable, Bold *P*-values means *P* < 0.05, significance level < 0.05

Before the intervention, high levels of stress (12.65; 12.25), anxiety (11.34; 11.02) and depression (10.08; 10.42) and low levels of self-confidence (1.31; 1.22) were observed in the intervention and control groups.

The results indicated a significant difference in the mean scores of self-confidence (*p* < 0.001), stress (*p* < 0.001), and academic progress (*p* < 0.001), between the intervention and control groups after the educational intervention. Furthermore, this difference was also statistically significant in the intervention group before and after the educational intervention (*p* < 0.05). However, there was no significant difference in the mean scores of anxiety and depression before and after the intervention, as well as in comparison with the control group (*p* > 0.05) (Table 2).

The results showed significant differences between the intervention and control groups. Post-intervention, the intervention group showed substantial increases in self-confidence (mean difference = 5.97, *p* < 0.001) and significant reductions in stress levels (mean difference = -3.22, *p* < 0.001). In contrast, minimal changes were observed in the control group for both self-confidence (mean difference = 0.057, *p* = 0.934) and stress levels (mean difference = 0.142, *p* = 0.656). While both groups exhibited decreases in anxiety and depression levels, these changes were not statistically significant (*p* > 0.05). Moreover, the intervention significantly improved academic progress in the intervention group compared to the control group (mean difference = 20.31, *p* < 0.001) (Table 2).

The ANCOVA test was used to compare the means of self-confidence, stress, anxiety, depression and academic progress in the two groups after adjusting the Pre-test as a covariate. Results showed there was a significant difference between the means in the self-confidence, stress and academic progress before and after intervention with adjusted pre- test score (before intervention) (Table 3).

**Table 3:** Comparison means of self-confidence, stress, anxiety, depression and academic progress in intervention and control groups after the intervention by adjusting the effect of the score before the intervention (Pre-test) using ANCOVA analysis

Variable	Source	Sum of squares	df	Mean Square	F	*P*-value	Partial Eta Squared	Noncent Parameter	Observed power
Self-confidence	Corrected Model	630.87	2	315.436	50.63	** < 0.001**	0.602	101.26	1.00
	Before intervention	0.871	1	0.871	0.140	0.710	0.002	0.140	0.066
	Group	629.07	1	629.07	100.97	** < 0.001**	0.601	100.97	1.00
	Error	417.41	67	6.230	NA	NA	NA	NA	NA
DASS Stress	Corrected Model	159.19	2	79.59	35.018	** < 0.001**	0.511	70.03	1.00
	Before intervention	4.67	1	4.67	2.05	0.156	0.030	2.05	0.293
	Group	158.78	1	158.78	69.85	** < 0.001**	0.510	69.85	1.00
	Error	152.29	67	2.27	NA	NA	NA	NA	NA
DASS Anxiety	Corrected Model	6.74	2	3.37	0.796	0.445	0.023	1.59	0.181
	Before intervention	6.04	1	6.04	1.42	0.236	0.021	1.42	0.218
	Group	1.00	1	1.00	0.238	0.627	0.004	0.238	0.077
	Error	283.83	67	4.23	NA	NA	NA	NA	NA
DASS Depression	Corrected Model	9.89	2	4.94	1.29	0.281	0.037	2.58	0.271
	Before intervention	1.66	1	1.66	0.436	0.511	0.006	0.436	1.00
	Group	7.23	1	7.23	1.88	0.174	0.027	1.88	0.273
	Error	256.38	67	3.82	NA	NA	NA	NA	NA
Academic progress	Corrected Model	8529.68	2	4664.84	10.96	** < 0.001**	0.247	21.92	0.989
	Before intervention	15.62	1	15.62	0.040	0.842	0.001	0.04	0.054
	Group	8415.61	1	8415.61	21.63	** < 0.001**	0.244	21.630	0.996
	Error	26,067.40	67	389.06	NA	NA	NA	NA	NA

Adjusted variables: Self-confidence, Stress, perceived Anxiety, Depression and Academic progress (Pre-test)

Bold *P*-values means *P* < 0.05, significance level < 0.05

The results of the univariate linear regression analysis showed that self-confidence and stress are associated with academic progress (*p* < 0.05) (Table 4). Additionally, the results of the multiple regression analysis revealed that for a one-unit increase in the stress score, the mean academic progress score decreases by 0.520 (B = -0.520, *P* < 0.001). Furthermore, for a one-unit increase in age, the mean academic progress score increases by 0.220(B = 0.220, *P* = 0.029). Moreover, students whose fathers have university education have, on mean, a higher academic progress score compared to students whose fathers are illiterate, with an increase of 0.212 for each unit difference in paternal education level (B = 0.212, *P* = 0.036). According to the multiple regression model, 33.4% of the variations in academic progress can be predicted by stress, age, and father’s education (Table 4).

**Table 4:** Factors affecting academic progress using the univariable and multivariable linear regression model. Model-based on 70 observations, adjusted R-squared = 33.4% for final model, *p* = 0.001

Variable	Category	Univariable				Multivariable			
		B	SE	Standardized Coefficients Beta	*P*-value**	B	SE	Standardized Coefficients Beta	*P*-value**
Age	NA	1.88	1.02	0.218	0.070	1.90	0.852	0.220	**0.029**
Self-confidence	NA	1.87	0.658	0.327	**0.006**	-	-	-	-
Stress	NA	-5.36	1.10	-0.509	** < 0.001**	-5.47	1.06	-0.520	** < 0.001**
Anxiety	NA	-0.697	1.32	-0.604	0.599	-	-	-	-
Depression	NA	-1.608	1.36	-0.141	0.244	-	-	-	
Gender	Male	Ref	-	-	-	-	-	-	-
	Female	-9.36	5.26	-0.218	0.070	-	-	-	-
Mother's education	Illiterate	Ref	-	-	-	Ref	-	-	-
	Elementary	5.40	6.53	1.0	0.411	-	-	-	-
	Secondary	-2.78	7.69	-0.044	0.719	-	-	-	-
	Diploma	6.49	5.75	0.136	0.263	9.04	4.87	0.189	0.068
	University	-11.78	8.35	-0.169	0.163	-	-	-	-
Father's education	Illiterate	Ref	-	-	Ref	Ref	-	-	-
	Elementary	-7.911	6.67	-0.142	0.240	-	-	-	-
	Secondary	0.667	8.98	0.009	0.941	-	-	-	-
	Diploma	-0.163	5.88	-0.003	0.978	-	-		-
	University	10.39	6.81	0.182	0.132	12.11	5.66	0.212	**0.036**
Family economic	Weak	-6.68	6.11	-0.131	0.278	-		-	-
	Medium	-0.536	5.42	-0.012	0.922	-		-	-
	Good	Ref	-	-	-	-	-	-	-

Variables entered in the multivariable Model: Age, Self-confidence, DASS Stress, DASS Anxiety, DASS Depression, gender, Mother's education, Father's education, Family economic

“- “: Not applicable, *Univariable linear regression, **Multiple linear regression using backward technique, Bold *P*-values means *P* < .05, significance level < 0.05

## Discussion

This research was conducted to determine the effect of peer mentoring program on clinical academic progress and psychological characteristics of operating room students.

The results showed that before the educational intervention, there was no significant difference between the control and intervention groups in demographic variables, academic progress, self-confidence, stress, anxiety and depression. It is noteworthy that according to the regression analysis, students whose fathers had a university education had a higher academic progress score compared to students whose fathers were illiterate.

The results of the study before the intervention show a high level of stress, anxiety and depression and a low level of self-confidence in students. Mohammadi's study showed the mean situational anxiety scores of the operating room students to be at a medium–high level [33]. Of course, according to Findik's study, the stress level of nursing students was low on the first day of operating room practice. It was found that students use the self-confidence approach in dealing with stress [34]. According to Norouzi's study, insufficient skills of students in communicating with staff, discrimination between paramedical students and assistants, lack of practical prerequisite skills, weak supportive performance of instructors and psychological needs are among the stressful factors of operating room students [3]. According to the students, practice with the support of staff and instructors in clinical training leads to better training. Improper interaction between staff and students negatively affects the clinical education process [35, 36]. The results of Mohibi's research report the existence of discrimination as one of the main complaints of students in the clinical environment [37].

The results showed that training using the peer mentor method improved the mean scores of self-confidence, stress and academic progress variables in the intervention group after the educational intervention. Also, compared to the control group, the intervention group had achieved a significant improvement in the mentioned variables. In addition, the results showed that self-confidence and stress are related to academic progress, and as the stress score increases, the mean academic progress decreases. The results of Raymond's study showed that the implementation of the mentorship program was effective in reducing the stress and loneliness of first-year nursing students. In addition, an increase in their sense of self-efficacy and sense of psychological belonging was also reported [38]. According to Yoon's study, peer mentoring program increased students' self-confidence in basic nursing skills and critical thinking skills [39]. Considering that clinical educators play a fundamental role in controlling stress, creating a supportive environment and promoting students' self-confidence in the clinical learning environment [40], it seems that the use of students in the role of peer mentoring has been able to act as an important factor in increasing self-confidence, reducing stress and enjoying clinical experiences and thus improving their academic progress.

While in Walker's study, a significant reduction in the anxiety of a specific clinical situation was observed among nursing students who were guided by their peers [41], in the present study, no significant improvement was observed in the students' anxiety. It can be said that the special conditions of the operating room distinguish it from other clinical skills training departments, therefore peer training alone cannot be effective in reducing the anxiety of operating room students. Also, depression did not decrease significantly in any of the intervention and control groups. It should be said that anxiety and depression are more complex than stress and their reduction in operating room students requires the use of psychological interventions along with peer mentoring program.

Due to the limitation of the statistical population, sampling was not possible and the students were selected by census method. On the other hand, due to the special considerations of the operating room space, the implementation of the peer mentoring program faced limitations. Although the main teacher of the course was present in all the implementation sessions of the mentorship program, physicians and other clinical personnel did not trust the mentors to some extent.

## Conclusion

The results showed that the implementation of the peer mentoring program was effective in improving academic progress, self-confidence, and reducing the stress of operating room students. Therefore, this educational method can be used in addition to the usual methods to improve the education of operating room students.

Of course, the use of this training method could not be effective in reducing anxiety and depression, which can be aggravated as a result of working in the tense environment of the operating room, and it seems necessary to conduct more investigations in this field.

## Data Availability

The datasets generated and analyzed during the current study are not publicly available because they contain raw data from study participants, and sharing these data requires participants' permission. But are available from the corresponding author on reasonable request.
